# Optogenetic stimulation of inferior colliculus neurons elicits mesencephalic locomotor region activity and reverses haloperidol-induced catalepsy in rats

**DOI:** 10.1038/s41598-025-96995-4

**Published:** 2025-04-12

**Authors:** José A. Pochapski, Jan Franke, Wolfgang Kruse, Ralf Jacob, Stefan Herlitze, Claudio Da Cunha, Rainer K. W. Schwarting, Liana Melo-Thomas

**Affiliations:** 1 Pharmacology Department, Federal University of Parana, Curitiba, Brazil; 2 Behavioral Neuroscience, Experimental and Biological Psychology, Philipps-University of Marburg, Marburg, Germany; 3 General Zoology and Neurobiology, Ruhr-University Bochum, Bochum, Germany; 4 Department of Cell Biology and Cell Pathology, Philipps-University Marburg, Marburg, Germany; 5 Biochemistry Department, Federal University of Parana, Curitiba, Brazil; 6 Center for Mind, Brain, and Behavior (CMBB), Marburg, Germany; 7 Behavioral Neuroscience Institute (INeC), São Paulo, Brazil

**Keywords:** Paradoxical kinesia, Inferior colliculus, Mesencephalic locomotor region, Catalepsy, Parkinson’s disease, Neuroscience, Physiology

## Abstract

The inferior colliculus (IC) represents a pivotal midbrain area involved in processing sensory/emotional facets of auditory stimuli, potentially influencing motor responses. Our prior investigations showed that electric or chemical stimulation of the IC ameliorates haloperidol-induced catalepsy, a manifestation of Parkinsonism in animals. We hypothesized that this amelioration stems from a sensory-motor gating mechanism via IC activation of the mesencephalic locomotor region (MLR). Aiming to investigate this hypothesis, we performed IC optogenetic stimulation and electrophysiological recordings of MLR neuronal activity in anesthetized rats. Additionally, we examined whether optogenetic manipulation of IC could improve motor deficits and affect emotional states in awake rats. Electrophysiological data revealed an excitatory response in MLR neurons following IC optogenetic stimulation, with a longer onset latency in MLR neurons suggesting synaptic modulation from IC to MLR. Behavioral results showed that IC optogenetic stimulation improved haloperidol-induced motor deficits without affecting emotional state or basal locomotor activity.

**Supplementary Information:**

The online version contains supplementary material available at 10.1038/s41598-025-96995-4.

## Introduction

The classic motor symptoms of Parkinson’s disease (PD), akinesia, bradykinesia, tremor, rigidity and postural instability, are associated with the degeneration of dopaminergic neurons in the substantia nigra pars compacta (SNc) and a decline in caudate-putamen dopamine levels. Haloperidol, clinically an antipsychotic drug, operates mainly by blocking dopaminergic receptors within the nigrostriatal pathway, leading to adverse motor effects^1^. In rodents, haloperidol induces a state of catalepsy characterized by the animals remaining immobile e.g., when positioned with the fore-paws on a horizontal bar (Sanberg, 1980). Hence, the bar test can be used to investigate the anti-parkinsonian efficacy of novel treatments to reverse haloperidol-induced catalepsy^2–4^. Studies conducted in our laboratory have revealed that both systemic and intrastriatal haloperidol-induced catalepsy in rats can be significantly reduced by electrical or chemical stimulation of the inferior colliculus (IC), a midbrain structure traditionally associated with auditory processing^5–10^. We have hypothesized that the reversal of haloperidol-induced catalepsy in rats results from sensory-motor gating involving the activation of mesencephalic locomotor region (MLR) by the IC^11^.

Indeed, emerging evidence suggests that some of the motor deficits observed in PD can be attributed to changes in the MLR^12^, leading clinicians to explore the effect of deep brain stimulation of the MLR as a potential treatment approach to enhance locomotor function in PD patients^13^. The MLR is a brainstem area defined functionally as a mesencephalic region in which electrical stimulation induces a transition from a stationary state to walking, followed by running with a short latency^14,15^. The gait deficits of PD patients can be partially mediated by misbalance in the projections from the basal ganglia to the MLR^16^. That way, in the context of PD, the loss of dopaminergic neurons not only impacts the basal ganglia but has also significant effects on the function of the MLR^17–19^. Interestingly, in addition to receiving strong projections from the SNc^12,17,20^, the MLR also receives projections from the IC^21,22^. These projections may provide the MLR with auditory-emotional/motivational features leading to an additional movement gain important for motor improvement in Parkinsonism (for review see^11^).

We hypothesize that optogenetic activation of the IC could decrease haloperidol-induced catalepsy, and that this motor improvement depends on the activation of IC-MLR neurons. Aiming to test our hypothesis we used the optogenetic technique since it has the advantage of avoiding the stimulation of fibers of passage, as it happens with electrical stimulation, and offers the option to compare the effects of neural activation or inhibition. Aiming to investigate whether the IC projections can modulate specifically the activity in the MLR, we combined the optogenetic technique with electrophysiological recordings to assess the neuronal activity in the MLR during IC stimulation in anesthetized rats.

Furthermore, to provide a comprehensive understanding of the effects of optogenetic manipulation in the IC, we performed optogenetic stimulation or inhibition during behavioral tests. We aimed to investigate whether selective light activation of Channelrhodopsin (ChR2) or Archaerhodopsin-3 (Arch) opsins expressed in the IC could ameliorate motor deficits induced by haloperidol in awake rats. Building upon previous observations of explosive motor behavior and emotional responses—either aversive or anxiolytic—elicited by electrical or pharmacological stimulation of this structure^9,10,23–25^, we expanded our investigation to assess additional behavioral parameters such as distance traveled, grooming, and rearing behavior during optogenetic manipulation. Additionally, considering that the IC is part of the primary neural substrates for the integration of aversive states in the brain, and is where initial selectivity for communication sounds is formed^26–28^, we also evaluated whether IC optogenetic manipulation could induce aversive or appetitive effects as assessed by the number of 50- and 22-kHz ultrasonic vocalization (USV) emission, which are related to appetitive and aversive states, respectively (for review see^29^). This behavioral approach allowed us to evaluate the multifaceted impact of IC manipulation on motor function, emotional states, and communication through vocalizations.

## Results

### MLR neurons exhibited heterogeneous responses to IC optogenetic stimulation

**Fig. 1:**
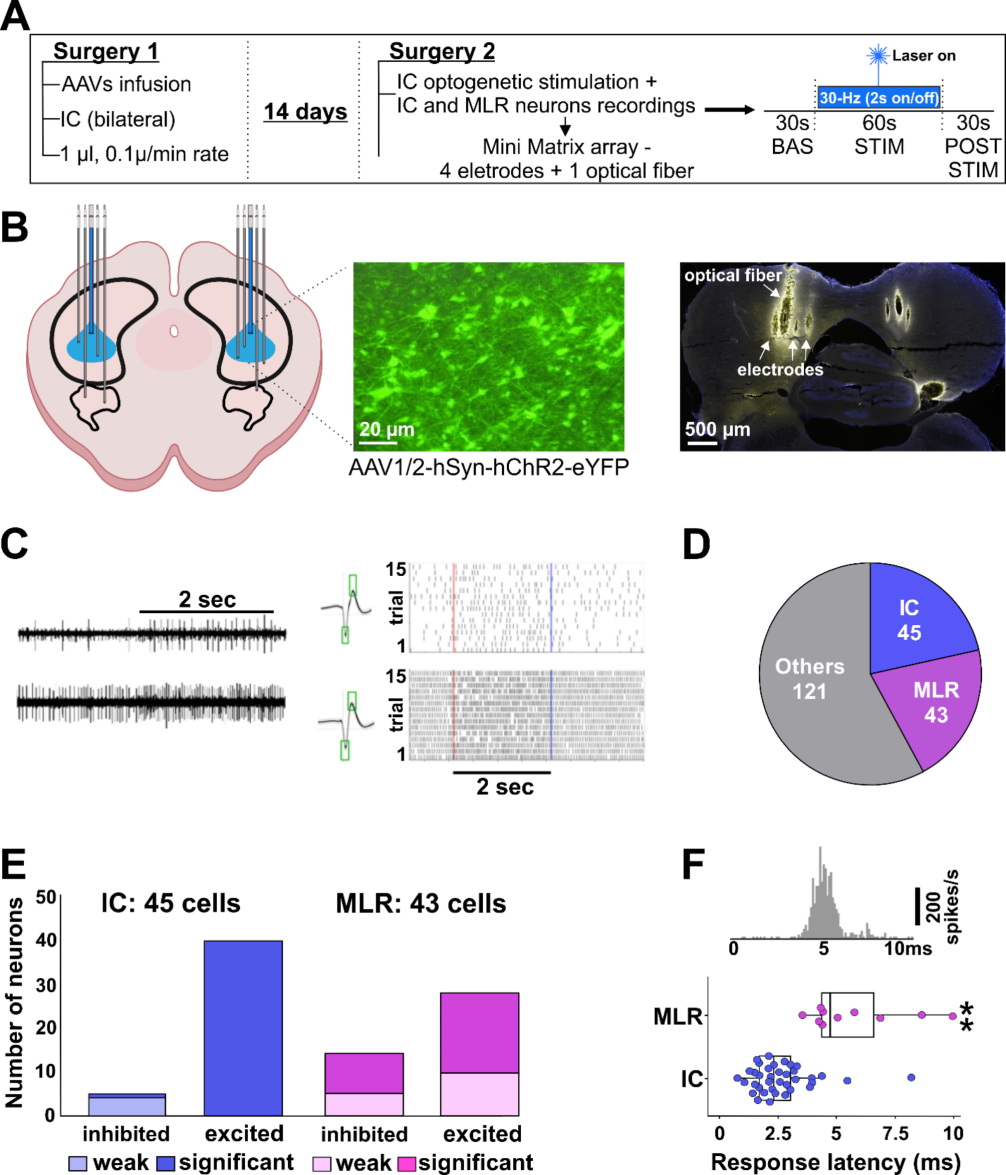
Intracollicular optogenetic stimulation associated with electrophysiological recordings of IC and MLR neurons activity. (**A**) Experiment timeline and experimental protocol summary. (**B**) Left—Illustrative representation of the IC and MLR neuronal activity recordings with 4 electrodes associated with IC optogenetic stimulation via a single optical fiber in the middle of the electrode array. Center—IC transfected neurons expressing the fluorescent marker after ChR2 AAVs injection. Right—Example of electrodes and optical fiber bilateral placements on IC. (**C**) Left—Example of two extracellular signals recorded simultaneously from two electrodes, Middle—averaged spike shapes detected from the adjacent raw signals. Right—Raster plots of spike activity during 15 repetitions of 2 s with 30 Hz light stimulation (2 ms pulses). Red and blue lines represent the stimulation onset and offset, respectively. (**D**) Total number of recorded neurons. From 209 cells recorded while the optical fiber was positioned in the IC, 45 were localized in the IC and 43 were from MLR. (**E**) Electrophysiological response of IC and MLR neurons during IC optogenetic stimulation. A total of 40 IC cells responded with a significantly increased spike rate during light application, whereas diminished activity was observed in 5 IC cells. In one IC cell, this inhibition reached significance (*p* < 0.05; T-test). In MLR, IC stimulation-induced inhibition was significant in 9 cells, and below significance in further 5 cells. A significant increase of spike rate during the 30 Hz stimulation was observed in 19 cells, and below significance in further 10 cells; (**F**) Top—Example for a neuron’s pulse response relative to pulse onset (at t = 0). This example shows an IC-recorded neuron with a peak onset latency of 3.23 ms. Bottom—Response latency for IC and MLR neurons. The IC neuron responded with a significantly shorter latency (2.32 ms) than MLR cells (4.74 ms, *P* < 0.005, T-test). Box plots indicate the median and quartiles. Individual subjects’ data are represented by circles. *P < 0.005 after IC and MLR comparison.

From four rats that received a ChR2 virus injection in the IC, the action potentials of 252 neurons were recorded during repetitive light stimulation. Histological verification of the depth position of the optical fiber and electrodes revealed that 209 cells were recorded while the tip of the optical fiber was placed in the IC (Supplementary Figs. S1 and S2). The other recordings were performed after the optical fiber had been driven to locations beyond the IC, mainly periaqueductal gray, MLR and below. From the 209 cells recorded during IC stimulation, 45 were histologically identified as located in the IC and 43 in the MLR (Fig. 1D). The remaining 121 cells were either from positions beyond the IC and MLR, or from the border region between IC and MLR and were excluded from further analysis.

The majority of cells recorded in the IC responded with a strong and significant response (t-test *p* ≤ 0.05 for each cell) to the 2 s stimulation periods (40/45 cells). From the remaining five cells, one cell showed a significant reduction in spike rate during light stimulation, and four cells were weakly suppressed, albeit not reaching the 5% level in the paired t-test after 15 repetitions (Fig. 1E, left).

Regarding MLR recordings, the activating effect of the light in the IC was also dominant (29/43 cells), but in 10 cells, only a weak, non-significant excitation emerged. In the remaining 14 cells, a reduced spike rate was observed during IC light stimulation. The inhibitory effect driven by the IC stimulation was significant (paired t-test, *p* < 0.05) in 9 MLR cells (Fig. 1E, right). In conclusion, the excitatory effect of the ChR2-mediated activation via focused light application to the IC was strongly dominant in the IC recordings, as expected. In the MLR, excitatory effects were observed in 67% of cells, and weak modulations that did not reach statistical significance in 15 repetitions (paired t-test, *p* ≥ 0.05) were more frequent.

In a fraction of the cells that showed a significant excitatory response to the light application, response to single pulses were detectable in pulse triggered averages related to the 900 pulses contained in the 30 Hz pulse trains (Fig. 1F, Top). Such pulse responses emerged in 37 out of 40 excitatory responding IC cells and 10 out of 19 excitatory responding MLR cells. The onset latency of the pulse response was significantly longer in MLR (median: 4.74 ms) than in the IC (median: 2.32 ms; t-test *p* < 0.005; Fig. 1F, Bottom).

### Optogenetic manipulation of the inferior colliculus did not affect locomotor activity and ultrasonic vocalizations

**Fig. 2:**
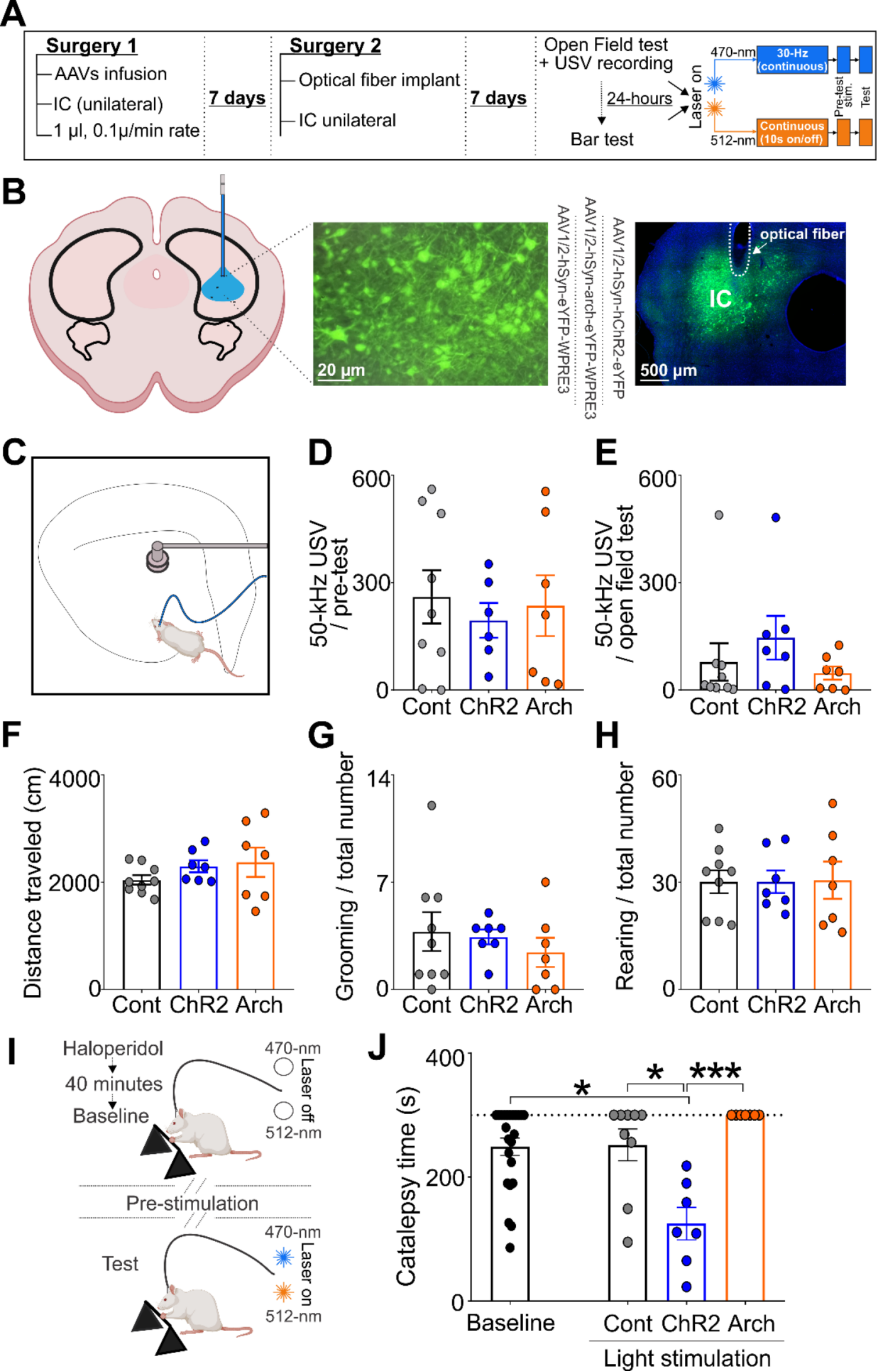
Optogenetic manipulation of IC during behavioral tests. (**A**) Experiment timeline and experimental protocol summary; (**B**) Left—Illustrative representation of the optogenetic manipulation in the IC. Center—IC transfected neurons expressing the fluorescent marker after the control (*n* = 9), Arch (*n* = 7), or ChR2 (*n* = 7) AAVs injection. Right—Example of an optical fiber placement on IC. (**C**) An illustrative depiction of the open field test showing the positioning of the microphone at the center of the open field, with rats receiving light stimulation (light 470 or 512-nm light stimulation) via a patch cable connected to the implanted optical fiber. Rats were submitted to a pre-test stimulation for 10 min before the open field test. (**D**) Total number of 50-kHz USVs recorded during the pre-test stimulation protocol and (**E**) during the open field test. (**F**) Total locomotion (cm) traveled during the open field test. (**G**) Total number of grooming and (**H**) rearing behavior; (**I**) Illustrative representation of the experimental protocol during the bar test. The catalepsy time was measured during a baseline period (no light stimulation) and during a test period (light stimulation using a 473-nm or 512-nm stimulus for optogenetic stimulation and inhibition, respectively); (**J**) Catalepsy time during the bar test is represented by the step-down latency (s). Data are expressed as mean ± SEM. Individual subjects’ data are represented by circles. *P < 0.05 and ***P < 0.001 compared to the ChR2 group, Dunn’s post hoc test after Kruskal-Wallis ANOVA.

IC stimulation or inhibition did not produce significant effects on the traveled distance (Fig. 2F; F(2, 20) = 1.14, *p* = 0.33; one-way ANOVA), number of grooming (Fig. 2G; F(2, 20) = 0.45, *p* = 0.63; one-way ANOVA) and rearing events (Fig. 2H; F(2, 20) = 0.004, *p* = 0.99; one-way ANOVA). Furthermore, during the pre-test session, no significant group difference was observed in the number of 50-kHz (Fig. 2D; F(2, 19) = 0.19, *p* = 0.82; one-way ANOVA) and 22-kHz USVs (W = 0.17, *p* = 0.89; Kruskal-Wallis ANOVA; Supplemental Fig. S3). Similarly, during the test session, no significant differences were detected following IC optogenetic stimulation or inhibition in the number of 50-kHz calls (Fig. 2E; W = 2.95, *p* = 0.22; Kruskal-Wallis ANOVA) and 22-kHz calls (W = 1.68, *p* = 0.42; Kruskal-Wallis ANOVA; Supplementary Fig. S3).

### Optogenetic stimulation of the inferior colliculus reduces haloperidol-induced catalepsy

Next, we investigated whether IC optogenetic inhibition or excitation affected catalepsy time (see Fig. 2I). Analysis of catalepsy time during the baseline period revealed that all rats exhibited a catalepsy state above the cataleptic threshold, and no significant differences were observed among the groups at this stage (Fig. 2J; F(2,20) = 0.33; *p* = 0.72). Therefore, the baseline data for the experimental groups were combined. Subsequently, after a pre-test light-stimulation period (470–512 nm), rats were re-exposed to the bar test. Under optogenetic manipulation, a significant difference in catalepsy time was observed (Fig. 2J; W = 15.59, *p* < 0.001; Kruskal-Wallis ANOVA). Dunn’s test showed that the ChR2 group exhibited a significantly lower latency to step down compared to the baseline period (*p* = 0.01). Furthermore, rats in the ChR2 group displayed a significantly lower step-down latency compared to the control group (*p* = 0.02, Dunn’s test) and the Arch group (*p* < 0.001; Dunn’s test). No significant difference was observed in the control group between the baseline and light stimulation periods (*p* > 0.99). Optogenetic inhibition of the IC in the Arch group caused no significant difference in step-down latencies compared to the baseline period (*p* = 0.27) or compared to the control group (*p* = 0.74). Histological analyses showed that the optical fiber tips were correctly positioned in the IC (see Supplementary Figs. S2 and S4).

## Discussion

In this study, we used optogenetics to investigate how stimulation of the IC affects activity of the IC-MLR projections and impact behavior in rats. We tested whether optogenetic manipulation of the IC affects neural activity in both the IC itself and the MLR. Although IC optogenetic stimulation elicits inhibition of some MLR neurons, a predominant excitatory neural response in both IC and MLR neurons was observed. Considering that the MLR modulates different facets of movement, it would not be expected that IC activation would result in the activation of all MLR neurons. Instead, IC projections may selectively engage specific neuronal populations depending on the functional requirements of the motor task. The predominant excitatory effect observed in the IC recordings highlights the impact of local ChR2-mediated activation via focused light application. Notably, MLR-recorded neurons displayed an excitatory effect following IC stimulation, indicating that the influence of IC activity extended into the MLR. Importantly, the onset latency of the pulse response was found to be longer in MLR-recorded neurons compared to IC-recorded neurons. This result suggests that there is a modulation of synaptic transmission from neurons projecting from the IC to MLR, potentially accounting for the delayed response in MLR neurons. These findings have important implications for understanding the neural circuitry underlying the behavioral outcome observed in the present study. Previous studies using optogenetic manipulation have shown that stimulation of glutamatergic neurons in MLR induced locomotion in mice^17^. Specifically, distinct glutamatergic subpopulation of neurons in MLR receive strong inputs from IC, but also from periaqueductal gray, basal ganglia nuclei and several midbrain and medullary sensory-motor nuclei^17,20,21^. When properly stimulated, these projections elicit fast locomotion, escape response or exploratory locomotor behavior^21,30–33^ suggesting participation in locomotion control. The projections from the basal ganglia also suggests that dysregulation of glutamatergic neurons in MLR may play important roles in PD related locomotor disorders. Thus, stimulation of IC-MLR projections could potentially compensate for this dysregulation and improve catalepsy as demonstrated in the present study. We have suggested that such IC-MLR projections may also be involved in the motor improvement occurring during paradoxical kinesia induced by IC stimulation^11^. Indeed, in the search for treatments for motor deficits as observed in PD, it would be interesting to look outside the degenerated dopaminergic pathway of the basal ganglia. For instance, one of such structures could be the IC. Previous data from our group suggested that the IC is part of an alternative pathway activated to induce paradoxical kinesia in Parkinsonian rats^5–10^.

Additionally, we aimed to investigate whether optogenetic manipulation of the IC impacts rat behavior. We evaluated the effects of this stimulation on motor and emotional behavioral outcomes. The same stimulation frequency used for electrophysiological experiment was applied during these behavioral tests. In the present study, we applied the optogenetic technique to explore the impact of stimulating or inhibiting the IC on haloperidol-induced catalepsy. Although catalepsy time during baseline was similar for all groups, under optogenetic manipulation, we observed a significant difference in catalepsy time. Specifically, IC stimulation in the ChR2-expressing group led to a significant decrease in the latency to step down compared to the baseline period. Furthermore, ChR2-expressing rats displayed a significantly lower step-down latency compared to both the control-expressing and the Arch-expressing groups. For the first time, these results reveal that increasing IC activity through optogenetic stimulation can relieve haloperidol-induced catalepsy. In contrast, when the IC was optogenetically inhibited in the Arch-expressing group, the step-down latency in this group did not exhibit a significant difference compared to either the baseline period or the control group.

Furthermore, the present study sought to determine whether such manipulation could influence the emotional state of the rats. This investigation stemmed from the understanding that the IC is part of the brain aversive system due to its ability to elicit vigorous escape behaviors such as wild running, jumping, and flight in response to electrical stimulation at specific current thresholds^23–25^. However, it has also been demonstrated that, depending on the IC stimulation parameters, anxiolytic or appetitive effects can be induced^9,10^. Aiming to investigate whether IC optogenetic activation or inhibition might induce any behavioral responses, rats were placed in an open field under 470 or 512-nm light stimulation in the IC and the number of USV emissions, locomotor activity, grooming and rearing behaviors were analyzed. The same stimulation parameters were used for catalepsy and open-field tests. Here, it is important to highlight that the rats received no drug treatment during this test. Our findings revealed that no significant effect of optogenetic activation or inhibition of the IC was observed on 50-kHz or 22-kHz USVs, indicating that optogenetic manipulation in the IC was neither appetitive nor aversive. This investigation was also important because the IC is where excitation and inhibition interact to create frequency-modulated selectivity^34^ and initial selectivity for communication sounds develops^26–28^. Regarding the assessment of spontaneous locomotion activity during the open field test, our results indicated that neither IC optogenetic stimulation nor inhibition had a significant impact on locomotor activity. Additionally, there were no significant differences in the total numbers of grooming or rearing behaviors. Furthermore, no evidence of aversive behaviors such as flight, wild running, or jumping, which can occur in response to IC electrical stimulation^23–25^ were observed during the tests. Additionally, we analyzed classical measures of anxiety-related behavior, such as time spent and number of entries in the center of the open field. No significant statistical differences were observed following IC stimulation (ChR2) or IC inhibition (Arch) in either the number of entries into the central zone or the total time spent there (data not shown). Collectively, these findings provide evidence that optogenetic stimulation of the IC effectively ameliorates motor deficits induced by haloperidol, while leaving the emotional state and basal locomotor activity unaffected in rats. The optogenetic stimulation used in the present study is much more specific compared to the electrical or chemical stimulation employed in our previous studies, and may have selectively activated a descending lower-level motor pathway, which was sufficient to alleviate haloperidol-induced catalepsy without involving emotional processing. However, these results do not rule out the possibility that motor improvements induced by IC stimulation may involve the activation of an auditory/emotional/motor pathway as extensively demonstrated in our earlier studies^5–11^. Importantly, the present results have clinical relevance, as IC activation could potentially be used to enhance motor performance in patients with PD or other motor deficits without impacting the patient’s emotional state.

Previously, we had demonstrated that IC electrical or chemical stimulation improved motor deficits in the context of paradoxical kinesia in rats treated with haloperidol^5–11^. Paradoxical kinesia, observed in individuals with PD^35^, entails a sudden improvement in complex movements, such as walking, dancing or ride a bicycle^36–38^, triggered by specific auditory or visual cues^39^. Although the neural basis of this phenomenon remains unknown, our experimental data suggest that in order for it to occur, the brain activates alternative pathways that bypass the defective basal ganglia in PD patients. We hypothesized that alternative pathways activated to ensure this motor improvement might, at least in part, involve projections from the IC to the MLR, both of which are situated outside the basal ganglia.

The present study provides insights into the neural interactions between the IC and MLR regions, shedding light on the mechanisms that underlie the behavioral outcomes observed following IC stimulation. We demonstrated that IC optogenetic stimulation can induce a robust excitatory response on MLR neurons, and can also reduce catalepsy in rats treated with systemic haloperidol without affecting basal locomotor and exploratory activity or inducing substantial emotional changes in undrugged rats, as indicated by the USV analysis. The present study contributes to our understanding of the neural circuits involved in motor control and may have implications for the development of novel therapeutic interventions for motor-related disorders. Further research is warranted to explore the specific mechanisms underlying these effects and their clinical relevance.

## Online methods

### Animals

Adult male Wistar rats (*n* = 35, Charles River Laboratories Germany GmbH, Köln) weighing between 270 and 320 g on the day of surgery were used in two distinct experiments. Out of these, four rats were used for electrophysiology and 31 rats were used for behavioral experiments. Behavioral data from eight rats were excluded due to misplacement of the optical fiber or low levels of viral expression. Rats were housed in Plexiglas cages in groups of three per cage and had *ad libitum* access to water and food (room temperature, 20 ± 2 °C; humidity, 55 ± 5%; 12 h light/dark cycle). After surgery, rats were kept individually for one day and were later housed in pairs in Macrolon type III cages with extra high acrylic covers (length, 22 cm; width, 38 cm; height, 38 cm). All protocols were performed in accordance with the EU regulations and approved by the local ethics committee (TVA G11-2021, Regierungspräsidium Giessen). The present study is reported in accordance with ARRIVE guidelines.

### Viral vectors and drugs

The following adeno-associated viruses (AAV) were used: AAV1/2-hSyn-hChR2(H134R)-eYFP (titer 6,90 × 1012 vg/ml), AAV1/2-hSyn-arch-eYFP-WPRE3 (titer 3,57 × 1012 vg/ml) and the control AAV1/2-hSyn-eYFP-WPRE3 (titer 5,14 × 1012 vg/ml) obtained from Charité viral core facility (Berlin, Germany). Catalepsy was induced by intraperitoneal injection of haloperidol (0.5 mg/kg, diluted in saline solution to 1 ml/kg vol., Janssen Pharmaceutica, Belgium).

### Surgery

#### Stereotaxic surgery for viral injection and analgesia regimen

To habituate the rats to the experimenter and the post-surgery analgesia procedures, three days prior to the stereotaxic surgery, rats were handled daily and received oral administration of meloxicam (0.5 mg/kg; p.o.). On the day of surgery, rats were anesthetized with isoflurane (2–3% Baxter, Germany) and placed in a stereotaxic frame (TSE Systems, Germany). Ophthalmic ointment Bepanthen (Bayer Vital, Germany) was applied to prevent eye drying. Then, each rat received a subcutaneous injection of 0.5 ml of local anesthetic (Xylocaine 2% with Adrenaline 1:100,000) at the incision site immediately before surgery. Viral microinjections were delivered using an injection cannula (30-gauge stainless steel) connected to a 10 µl Hamilton syringe by a polyethylene tube. Rats assigned to electrophysiology experiments received microinjections with 1 µl of either AAV-ChR2 or AAV-Control bilaterally into the IC in a 0.1 µl/min rate (Fig. 1A) using a microinjection pump (model sp101i, WPI, UK) according to the following coordinates relative to bregma: anteroposterior, 8.4 mm; mediolateral, ±1.5 mm; dorsoventral, 4.5 mm (from the dura mater^40^). The infusion cannula was left in place for an additional 10 min to allow diffusion and was slowly removed thereafter. Rats assigned to behavioral experiments underwent the same microinjection procedure except that either AAV-ChR2 (*n* = 7), AVV-Arch03 (*n* = 7) or AAV-Control (*n* = 9) were microinjected unilaterally into the IC (Fig. 2A, B). After injection, the scalp incisions were sutured. Post-operative care included buprenorphine (0.05 mg/kg s.c.; Titolare A.I.C., UK) injected 30 min before and 6 h after surgery, and twice on the day after surgery. Meloxicam (0.5 mg/kg; p.o, Metacam^®^, Boehringer Ingelheim, Germany) was administered every 12 h for 72 h after surgery. Body weights and general health status were monitored daily up to the end of the behavioral experiments.

### Electrophysiological experiments

For the simultaneous optical stimulation and electrophysiological recordings fourteen days after the virus injection, the animals were anesthetized and a second stereotaxic surgery was conducted (for details see above). Using a dental drill, the scalp was opened again to expose, reopen and slightly enlarge the boreholes from the prior virus injection. Next, microelectrodes were slowly moved into the brain tissue. Extracellular activity was recorded bilaterally, but not simultaneously, using the Mini Matrix system (Mini-Matrix, Thomas Recording GmbH, Giessen, Germany), which was customized to have an array of 4 single electrodes (impedance 0.3 to 2.3 MΩ) placed in a single row (Fig. 1B), with a light-conducting optical fiber positioned in the middle of the row. The guide-tube diameter of the Mini-Matrix was 312 μm, resulting in a total horizontal distance of 1248 μm between the two outermost electrodes. In the electrophysiological experiments, the optogenetic procedure was based on the technique described by Kruse et al.^41^.

The optical fiber (Thomas Recording GmbH, Giessen, Germany) had an outer diameter of 120 μm with a conically designed tip^41^. Light stimulation was applied through the optical fiber, with a 470 nm laser diode controlled by a LED light source control unit (Thomas Recording GmbH, Giessen, Germany). Before the insertion into the brain tissue, the light intensity from the optical fiber tip was measured using a laser power meter (PM160, Thorlabs, New Jersey, USA) and the obtained power was in a 500 to 750 µW range.

Light pulses with a duration of 2 ms were delivered with 30 Hz for 2 s, followed by 2 s without light. These 4-second sequences were repeated 15 times, for a total of 60 s. The temporal sequence of the laser light was controlled via custom-made software implemented in Matlab (Version 2019a, Mathworks, www.mathworks.com) and by using Arduino micro-computers (Arduino, Italy) to generate the 5 V trigger pulses to control the LED control unit. Additionally, TTL signals were generated during the optogenetic stimulation protocol and were sampled together with the electrophysiological data. Before and after the 60 s stimulation protocol, additional 30 s of baseline activity were recorded before new activity was searched for by driving one or multiple electrodes of the multi-electrode system to new depth positions.

#### Data acquisition and analysis

The extracellular signals were pre-amplified (gain = 19; Mini-Matrix) and main-amplified (gain = 250 to 500; MAF-05; Thomas Recording GmbH, Gießen, Germany), filtered (band pass, 500 Hz to 20 kHz), displayed in real-time and stored on a PC via a data acquisition system with 32 kHz (MC-Rack software, Version 4.6.2, www.multichannelsystems.com, Multichannel Systems MCSGmbH, Reutlingen, Germany). For the detection of action potentials, the stored signals were analyzed offline with custom-made software written in Matlab^41^. The spike sorting was based on a time/amplitude window discrimination algorithm. Signals were selected for spike detection when the spikes’ average peak to peak amplitude of spikes was at least three times the background signal variability. Spike rates during 2 s of 30 Hz light pulse trains were compared with 2 s reference periods without light between pulse trains (Fig. 1C). Cell activity was considered significantly modulated by light when a paired t-test based on 15 light applications yielded a p-value below 5%.

Cell activity significantly excited by light was tested for the average response latency relative to the light pulses. The average response to the 900 light pulses was calculated (Fig. 1F, Top). When a clear pulse response was obtained, the onset latency of the light pulse response was detected based on the time when the response peak crossed the threefold baseline variability. The outcome of the algorithm written in Matlab was graphically visualized, supervised by the user and manually corrected, if needed.

### Behavioral experiment

#### Optical fiber implantation

One week after viral injection, a second stereotaxic surgery was performed as described above, except that an optical fiber (outer diameter of 120 μm, numerical aperture 0.50; Thomas Recording GmbH, Germany; conically designed tip; see^41^ held in a 2.5 mm ceramic ferrule) was implanted unilaterally (300 μm above the viral injection site) for the stimulation of the IC transfected neurons (Fig. 2A and B). The optical fiber and four screws fixed to the skull were covered using adhesive (Duo-Link Universal, adhesive cementation System - Bisco) and dental acrylic resin. Post-operative care was identical to the procedure described for the first surgery (for details see above). The behavioral tests started one week after the second stereotaxic surgery.

#### Optogenetic manipulation of the inferior colliculus during behavioral tests

For assessing the effect of IC optogenetic manipulation on the behavior of freely moving rats, a patch cable (100 cm long, Thorlabs, USA) attached to a rotary joint (FL2, Thorlabs, USA) was connected to the ferrule in the implanted optical fiber through a ceramic mating sleeve. The optical stimulation was performed using an LED source (470 nm, blue or 512 nm, yellow, Thomas Recording GmbH, Giessen, Germany). During the behavioral tests, optical activation of transfected neurons was conducted according to the following protocol: For the ChR2 group (*n* = 7), we utilized continuous 30 Hz stimulation (the same frequency employed in the electrophysiological experiments). For the Arch group (*n* = 7), a continuous 10-second on/off stimulus was delivered. Rats injected with the control AAV (*n* = 9) were stimulated using 470-nm or 512-nm LED sources using the same protocols described for the ChR2 or Arch groups. Before the behavioral tests, the light intensities measured at the end of the patch cable matched those recorded for the electrophysiological experiments.

#### Open field test and USV recording

The effects of IC optogenetic manipulations on locomotion (i.e. distance traveled), rearing, and grooming were simultaneously recorded while rats freely explored an open field (60 cm width x 60 cm length x 40 cm height) under ambient red light (~ 28 lx; Fig. 2C). Prior to that, rats were handled and underwent habituation to the procedure of connecting the patch cable to the implanted optic fiber for three consecutive days in a standardized way (for 5 min each day).

On the test day, each rat was connected to the LED source and the control unit via the patch cable for continuous optogenetic stimulation with 470–512 nm light. They were placed in a polycarbonate cage (22 × 38 × 38 cm, with the floor covered in sawdust) for 10 min (pre-test session) and then moved to the open field to explore for 5 min (test session), all while under continuous light stimulation. The stimulation time used here is according to the protocol for electrical stimulation previously described by Ihme et al.^10^. Video recordings were captured by an overhead camera positioned 150 cm above the center of the open field. Analysis of locomotor activity was automatically performed using EthovisionXT software (Noldus, The Netherlands). The numbers of rearing and grooming events were manually scored.

Additionally, we recorded USV emission during IC optogenetic manipulation, throughout the pre-test stimulation session, and during the open field test using an ultrasonic condenser microphone (CM16, Avisoft Bioacoustics, Germany) mounted 50 cm above the center of the polycarbonate cage (for the pre-stimulation session) and above the center of the open-field apparatus. Fifty-kHz or 22-kHz calls were automatically identified using DeepSqueak 2.6.1 software^42^, and were manually accepted or rejected based on a previous study^43^.

#### Bar test

For the bar test, catalepsy was induced by haloperidol (0.5 mg/kg) diluted in physiological saline (0.9%) and administered i.p. in a volume of 1.0 ml/kg. Testing was performed on a squared arena^44^ (1 m^2^), elevated 50 cm above the floor under red light (~ 30 lx). Two cameras (Panasonic WVBP330/GE, Hamburg, Germany) were placed above (∼150 cm) and in front (∼40 cm) of the arena. A horizontal bar (8 cm height) was placed in the center of the arena. The bar test started with a baseline assessment. For that purpose, rats were placed with their forepaws on the bar 40 min after haloperidol injection. Catalepsy time was defined as the latency for both forepaws to step down from the bar and touch the floor. Catalepsy time was recorded with a cut-off time of 5 min. The baseline catalepsy time was taken only when a given rat remained at least 90 s on the bar. If a particular rat did not meet this criterion, testing was repeated after a 10 min interval, with a maximum of 5 repetitions allowed. The step-down-latency of the first successful trial was considered as baseline indicating the strength of drug-induced catalepsy. The IC light stimulation started immediately thereafter. For this purpose, the LED source patch cable was connected to the implanted ferrule, and the rats were individually placed into the polycarbonate cage with the floor covered in sawdust for the 10 min pre-test optical stimulation, as described above (see open field test). Subsequently, the rat´s forepaws were placed over the bar, and catalepsy time was recorded under IC optical intervention (Fig. 2I).

#### Data analysis

The behavioral data were tested for normality distribution using the D’Agostino-Pearson test. The data that presented a normal distribution were analyzed using a one-way ANOVA test followed by Tukey’s post hoc test. Data that did not present a normal distribution were analyzed using Kruskal-Wallis ANOVA followed by the Dunn’s test for multiple comparisons.

### Histological analysis

Following completion of the electrophysiological recordings and behavioral tests, rats were euthanized by an overdose of pentobarbital (Fagron GmbH & Co, Germany; 600 mg/kg). Once a deeply anesthetized state was confirmed, rats underwent transcardiac perfusion with saline (0.9%), followed by paraformaldehyde (4%, PFA) diluted in phosphate-buffered saline (PBS). Subsequently, the brain was carefully extracted and immersed in a cryoprotectant fixative solution (30% sucrose, 4% PFA solution) for three days. Thereafter, the brains were rapidly frozen and stored at – 80 °C. Coronal slices (50 μm) were obtained from the IC and MLR using a cryostat microtome (Model 1850, Leica). The slices containing the IC were mounted with Fluoromount mounting medium-DAPI (SIGMA Aldrich, Missouri, USA) and allowed to air-dry at room temperature. The positions of the optical fiber and electrodes, as well as the expression of fluorescent markers (EYFP) for the ChR2, Arch, and control groups were assessed using a fluorescence microscope (Leica DMi8, Germany) and compared with the brain atlas of Paxinos and Watson^40^.

## Electronic supplementary material

Below is the link to the electronic supplementary material.

Supplementary Material 1

Supplementary Material 2

## Data Availability

The datasets used and/or analysed during the current study is available from the corresponding author on reasonable request.

## References

[1] Farde, L. et al. Positron emission tomographic analysis of central D1 and D2 dopamine receptor occupancy in patients treated with classical neuroleptics and clozapine: relation to extrapyramidal side effects. *Arch. Gen. Psychiatry* **49**, 538–544 (1992). 1352677 10.1001/archpsyc.1992.01820070032005

[2] Sanberg, P. R. Haloperidol-induced catalepsy is mediated by postsynaptic dopamine receptors. *Nature* **284**, 472–473 (1980). 7189016 10.1038/284472a0

[3] Greco, B., Lopez, S., van der Putten, H., Flor, P. J. & Amalric, M. Metabotropic glutamate 7 receptor subtype modulates motor symptoms in rodent models of Parkinson’s disease. *J. Pharmacol. Exp. Ther.* **332** (3), 1064–1071 (2010). 19940105 10.1124/jpet.109.162115

[4] Reavill, C., Kettle, A., Holland, V., Riley, G. & Blackburn, T. P. Attenuation of haloperidol-induced catalepsy by a 5-HT2C receptor antagonista. *Br. J. Pharmacol.* **126** (3), 572–574 (1999). 10188965 10.1038/sj.bjp.0702350 PMC1565856

[5] Melo, L. L. et al. Coimbra, N. C. Glutamatergic neurotransmission mediated by NMDA receptors in the inferior colliculus can modulate haloperidol-induced catalepsy. *Brain Res.* **1349**, 41–47 (2010). 20558148 10.1016/j.brainres.2010.06.020

[6] Medeiros, P., Viana, M. B., Barbosa-Silva, R. C. & Tonelli, L. C. Melo-Thomas, L. Glutamatergic neurotransmission in the inferior colliculus influences intrastriatal haloperidol-induced catalepsy. Behav. *Brain Res.* **268**, 8–13 (2014). 10.1016/j.bbr.2014.03.027 24667361

[7] Tostes, J. G., Medeiros, P. & Melo-Thomas, L. Modulation of haloperidol-induced catalepsy in rats by GABAergic neural substrate in the inferior colliculus. *Neuroscience* **255**, 212–821 (2013). 24125891 10.1016/j.neuroscience.2013.09.064

[8] Melo-Thomas, L. & Thomas, U. Deep brain stimulation of the inferior colliculus: a possible animal model to study Paradoxical kinesia observed in some parkinsonian patients? *Behav. Brain Res.* **279**, 1–8. 10.1016/j.bbr.2014.10.035 (2015). 25446814 10.1016/j.bbr.2014.10.035

[9] Engelhardt, K. A., Marchetta, P., Schwarting, R. K. W. & Melo-Thomas, L. Haloperidol-induced catalepsy is ameliorated by deep brain stimulation of the inferior colliculus. *Sci. Rep.* **8**, 2216 (2018). 29396521 10.1038/s41598-018-19990-y PMC5797241

[10] Ihme, H., Schwarting, R. K. W. & Melo-Thomas, L. Low frequency deep brain stimulation in the inferior colliculus ameliorates haloperidol-induced catalepsy and reduces anxiety in rats. *PLoS ONE* **15**, e0243438 (2020). 33275614 10.1371/journal.pone.0243438 PMC7717509

[11] Melo-Thomas, L. & Schwarting, R. K. W. Paradoxical kinesia May no longer be a paradox waiting for 100 years to be unraveled. *Rev. Neurosci.* **34** (7), 775–799. 10.1515/revneuro-2023-0010 (2023). 36933238 10.1515/revneuro-2023-0010

[12] Ryczko, D. & Dubuc, R. Dopamine and the brainstem locomotor networks: from lamprey to human. *Front. Neurosci.* **11** 295. 10.3389/fnins.2017.00295 (2017). 28603482 10.3389/fnins.2017.00295 PMC5445171

[13] Plaha, P. & Gill, S. S. Bilateral deep brain stimulation of the pedunculopontine nucleus for Parkinson’s disease. *Neuroreport* **16**, 1883–1887 (2005). 16272872 10.1097/01.wnr.0000187637.20771.a0

[14] Shik, M. L., Orlovskii, G. N. & Severin, F. V. Organization of locomotor synergism. *Biofizika* **11**, 879–886 (1966). 6000596

[15] Shik, M. L., Severin, F. V. & Orlovskii, G. N. Control of walking and running by means of electric stimulation of the midbrain. *Biofizika* **11**, 659–666 (1966). 6000625

[16] Garcia-Rill, E. The basal ganglia and the locomotor regions. *Brain Res.* **396** (1), 47–63 (1986). 2871904

[17] Roseberry, T. K. et al. Cell-type-specific control of brainstem locomotor circuits by basal ganglia. *Cell* **1643**, 526–537 (2016). 10.1016/j.cell.2015.12.037 PMC4733247 26824660

[18] Stephenson-Jones, M., Samuelsson, E., Ericsson, J., Robertson, B. & Grillner, S. Evolutionary conservation of the basal ganglia as a common vertebrate mechanism for action selection. *Curr. Biol.* **21**, 1081–1091 (2011). 21700460 10.1016/j.cub.2011.05.001

[19] Kravitz, A. V. et al. Regulation of parkinsonian motor behaviours by optogenetic control of basal ganglia circuitry. *Nature* **466**, 622–626 (2010). 20613723 10.1038/nature09159 PMC3552484

[20] Ryczko, D. & Dubuc, R. The multifunctional mesencephalic locomotor region. *Curr. Pharm. Des.* **19**, 4448–4470 (2013). 23360276 10.2174/1381612811319240011

[21] Caggiano, V. et al. Midbrain circuits that set locomotor speed and gait selection. *Nature* **553** (7689), 455–460 (2018). 29342142 10.1038/nature25448 PMC5937258

[22] Melo-Thomas, L. et al. Lateralization in hemi-parkinsonian rats is affected by deep brain stimulation or glutamatergic neurotransmission in the inferior colliculus. *eNeuro* **9** (4), 2022. 10.1523/ENEURO.0076-22.2022 (2022). 10.1523/ENEURO.0076-22.2022 PMC9337613 35817565

[23] Melo, L. L. & Brandão, M. L. Role of 5-HT1A and 5-HT2 receptors in the aversion induced by electrical stimulation of inferior colliculus. *Pharmacol. Biochem. Behav.* **51**, 317–321 (1995). 7667347 10.1016/0091-3057(94)00387-x

[24] Melo, L. L., Cardoso, S. H. & Brandão, M. L. Antiaversive action of benzodiazepines on escape behavior induced by electrical stimulation of the inferior colliculus. *Physiol. Behav.* **51**, 557–562 (1992). 1326114 10.1016/0031-9384(92)90179-6

[25] Brandão, M. L., Troncoso, A. C., de Souza Silva, M. A. & Huston, J. P. The relevance of neuronal substrates of defense in the midbrain tectum to anxiety and stress: empirical and conceptual considerations. *Eur. J. Pharmacol.* **463**, 225–233 (2003). 12600713 10.1016/s0014-2999(03)01284-6

[26] Bauer, E. E., Klug, A. & Pollak, G. D. Spectral determination of responses to species-specific calls in the dorsal nucleus of the lateral lemniscus. *J. Neurophysiol.* **88**, 1955–1967 (2002). 12364521 10.1152/jn.2002.88.4.1955

[27] Casseday, J. H., Fremouw, T. & Covey, E. The inferior colliculus: A hub for the central auditory system. In *Integrative Functions in the Mammalian Auditory Pathway* (eds Oertel, D. et al.) Vol. 15 (Springer, 2002).

[28] Pollak, G. D., Xie, R., Gittelman, J. X., Andoni, S. & Li, N. The dominance of Inhibition in the inferior colliculus. *Hear. Res.* **274**, 27–39 (2011). 20685288 10.1016/j.heares.2010.05.010 PMC3762690

[29] Wöhr, M. & Schwarting, R. K. W. Affective communication in rodents: ultrasonic vocalizations as a tool for research on emotion and motivation. *Cell. Tissue Res.* **354**, 81–97 (2013). 23576070 10.1007/s00441-013-1607-9

[30] Costa, R. M. Plastic corticostriatal circuits for action learning: what’s dopamine got to do with it? *Ann. N. Y. Acad. Sci.* **1104**, 172–191 (2007). 17435119 10.1196/annals.1390.015

[31] Jordan, L. M., Liu, J., Hedlund, P. B., Akay, T., Pearson, K. G. & Descending command systems for the initiation of locomotion in mammals. *Brain Res. Rev.* **57**, 183–191 (2008). 17928060 10.1016/j.brainresrev.2007.07.019

[32] Grillner, S., Robertson, B. & Stephenson-Jones, M. The evolutionary origin of the vertebrate basal ganglia and its role in action selection. *J. Physiol.* **591**, 5425–5431 (2013). 23318875 10.1113/jphysiol.2012.246660 PMC3853485

[33] Friend, D. M. & Kravitz, A. V. Working together: basal ganglia pathways in action selection. *Trends Neurosci.* **37**, 301–303 (2014). 24816402 10.1016/j.tins.2014.04.004 PMC4041812

[34] Pollak, G. D. The dominant role of Inhibition in creating response selectivities for communication calls in the brainstem auditory system. *Hear. Res.* **305**, 86–101. 10.1016/j.heares.2013.03.001 (2013). 23545427 10.1016/j.heares.2013.03.001 PMC3778109

[35] Souques, M. A. Rapport Sur les syndromes parkinsoniens. *Rev. Neurol.* **37**, 534–573 (1921).

[36] Nombela, C., Hughes, L. E., Owen, A. M. & Grahn, J. A. Into the Groove: can rhythm influence Parkinson’s disease? Neurosci. *Biobehav. Rev.* **37**, 2564–2570 (2013). 10.1016/j.neubiorev.2013.08.003 24012774

[37] Snijders, A. H., Toni, I., Ruzicka, E. & Bloem, B. R. Bicycling breaks the ice for freezers of gait. *Mov. Disord.* **26** (3), 367–371 (2011). 21462254 10.1002/mds.23530

[38] Rodríguez-Quiroga, A. S., Rey, R. D., Arakaki, T. & Garretto, N. S. Dramatic improvement of parkinsonism while dancing Tango. *Mov. Disord Clin. Pract.* **1** (4), 388–389 (2014). 33999978 10.1002/mdc3.12067 PMC6353319

[39] Suteerawattananon, M., Morris, G. S., Etnyre, B. R., Jankovic, J. & Protas, E. J. Effects of visual and auditory cues on gait in individuals with Parkinson’s disease. *J. Neurol. Sci.* **219** (1–2), 63–69 (2004). 15050439 10.1016/j.jns.2003.12.007

[40] Paxinos, G. & Watson, P. *The Rat Brain in Stereotaxic Coordinates* 3 edn (Academic, 2007). 10.1016/0165-0270(80)90021-7 6110810

[41] Kruse, W. et al. Optogenetic modulation and multi-electrode analysis of cerebellar networks in vivo. *PLoS ONE* **9** (8), e105589. 10.1371/journal.pone.0105589.eCollection (2014). 25144735 10.1371/journal.pone.0105589 PMC4140813

[42] Coffey, K. R., Marx, R. E. & Neumaier, J. F. DeepSqueak: a deep learning-based system for detection and analysis of ultrasonic vocalizations. *Neuropsychopharmacology* **44** (5), 859–868 (2019). 30610191 10.1038/s41386-018-0303-6 PMC6461910

[43] Sangarapillai, N., Ellenberger, M., Wöhr, M. & Schwarting, R. K. W. Ultrasonic vocalizations and individual differences in rats performing a Pavlovian conditioned approach task. *Behav. Brain Res.* **398**, 112926. 10.1016/j.bbr.2020.112926 (2021). 33049281 10.1016/j.bbr.2020.112926

[44] Tonelli, L. C., Wöhr, M., Schwarting, R. & Melo-Thomas, L. Awakenings in rats by ultrasounds: A new animal model for Paradoxical kinesia. *Behav. Brain Res.* **337**, 204–209. 10.1016/j.bbr.2017.09.021 (2018). 28916501 10.1016/j.bbr.2017.09.021

