# Supplementary material for: Optogenetic stimulation of inferior colliculus neurons elicits mesencephalic locomotor region activity and reverses haloperidol-induced catalepsy in rats
Source: Sci Rep. 2025 Apr 12;15:12649. doi: 10.1038/s41598-025-96995-4 (PMC11993560; doi:10.1038/s41598-025-96995-4)

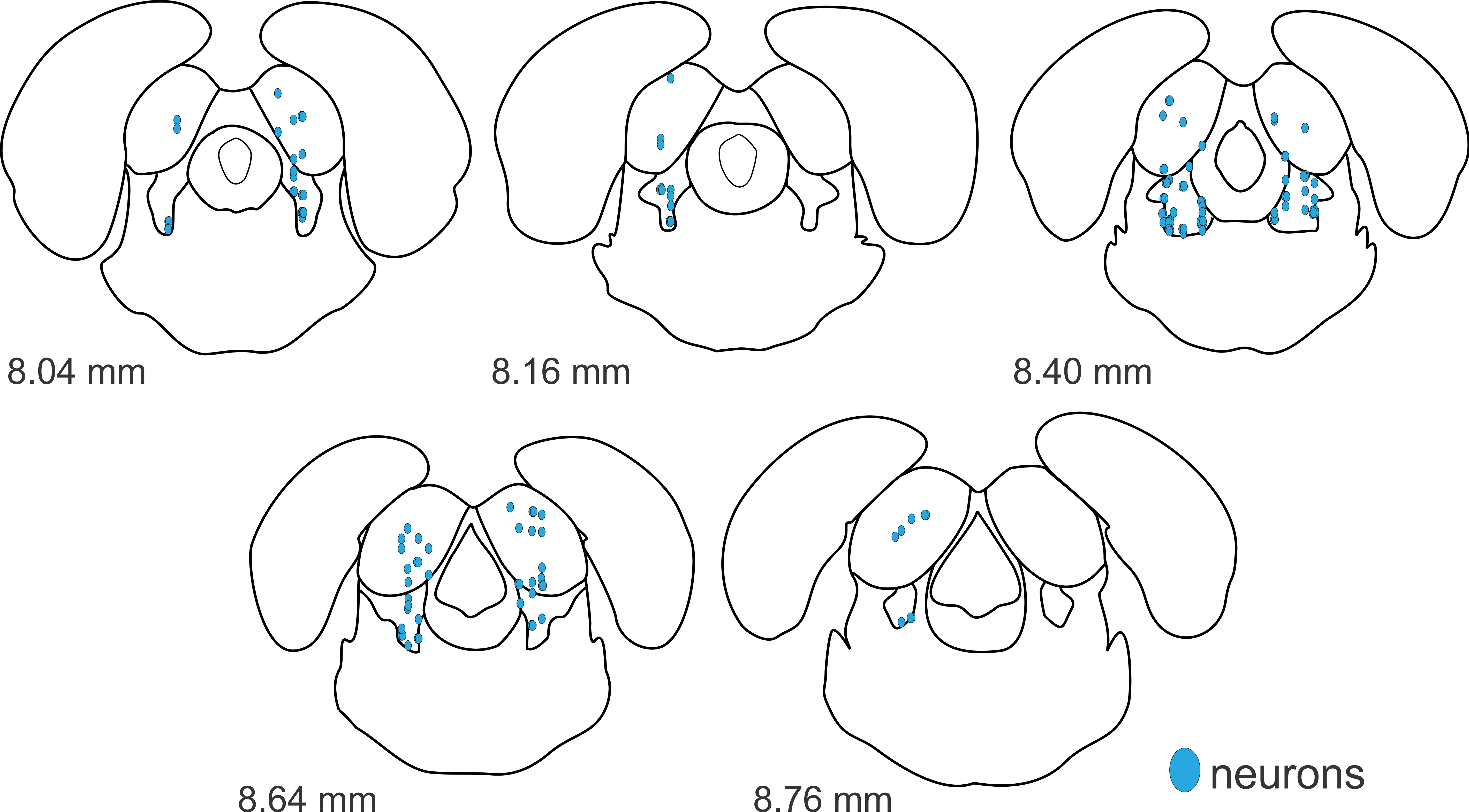
 Fig S1_supplementary figure 1

Fig S2_supplementary figure 2


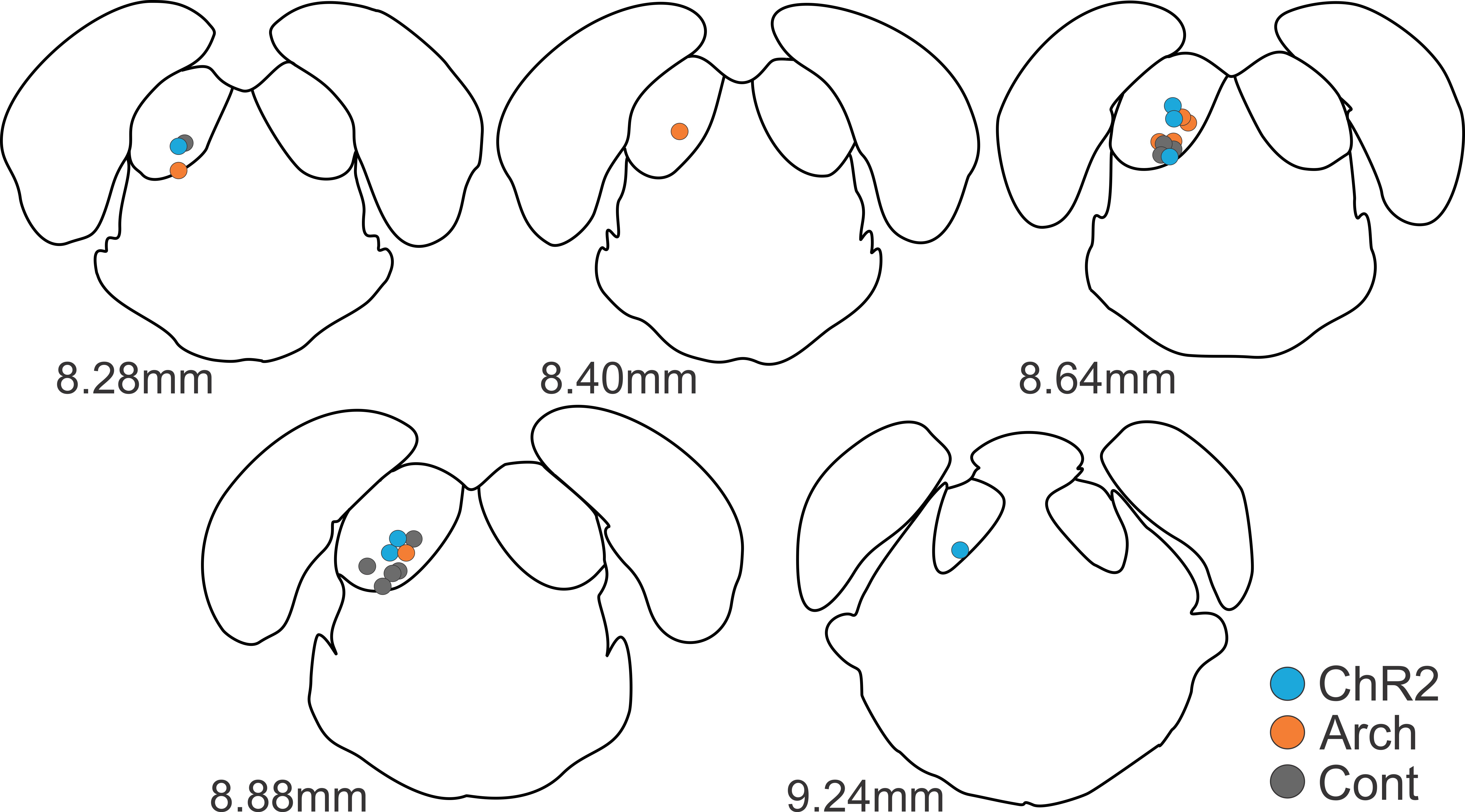


Fig S3_supplementary figure 3
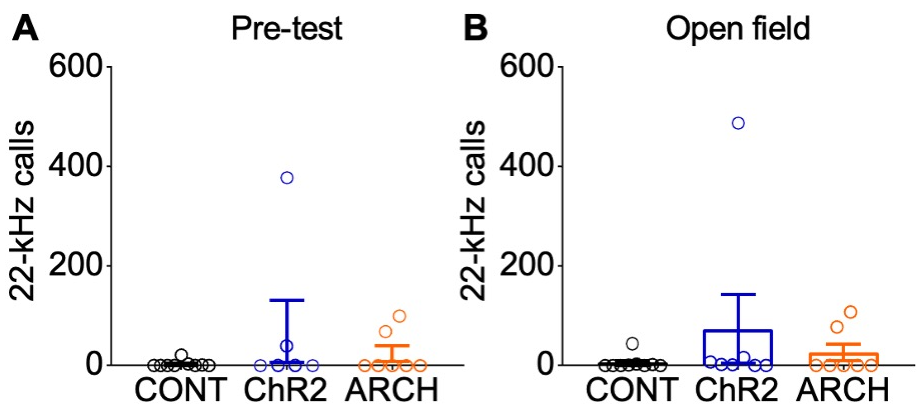


Fig S4_supplementary figure 4


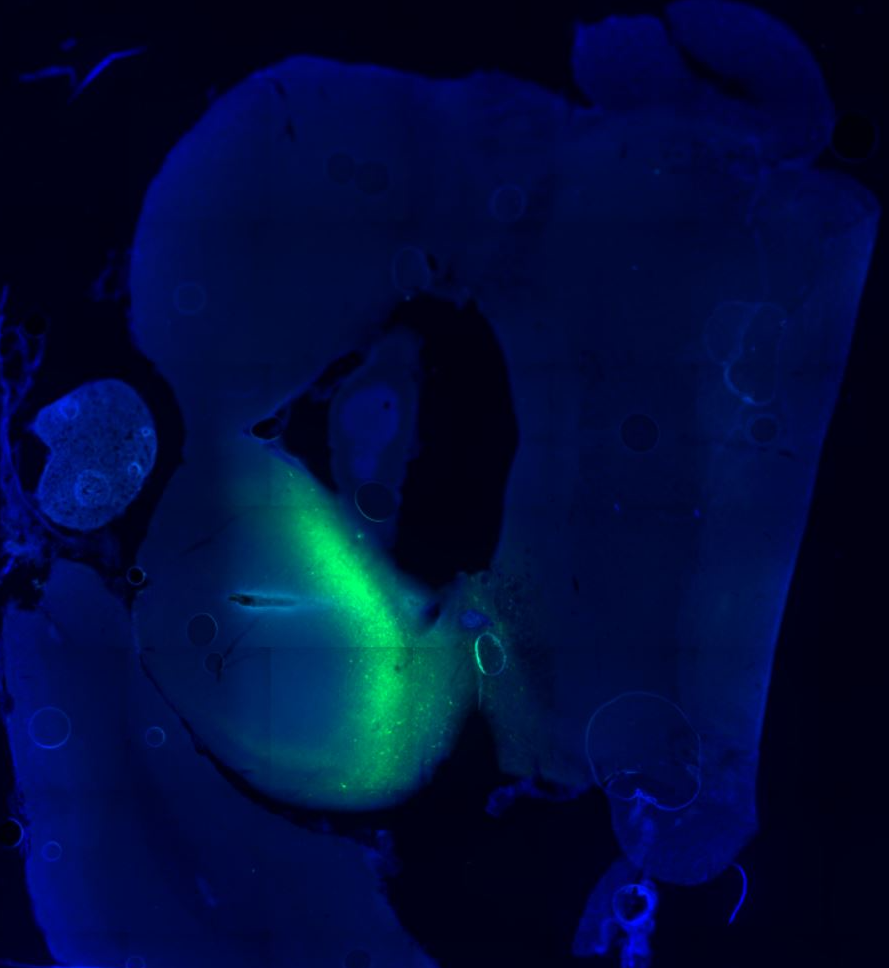

Supplement: Supplementary file 1 — Supplementary Material 1 [file 41598_2025_96995_MOESM1_ESM.docx]
