# Supplementary material for: Optogenetic stimulation of inferior colliculus neurons elicits mesencephalic locomotor region activity and reverses haloperidol-induced catalepsy in rats
Source: Sci Rep. 2025 Apr 12;15:12649. doi: 10.1038/s41598-025-96995-4 (PMC11993560; doi:10.1038/s41598-025-96995-4)
Supplement: Supplementary file 2 — Supplementary Material 2 [file 41598_2025_96995_MOESM2_ESM.docx]

**Legends for Supplemental Material**

**Supplemental Figure S1. Histological representation of the recording placements.**

**Supplemental Figure S2. Histological representation of the optical fiber tip placements for the behavioral experiment.**

**Supplemental Figure S3. Total number of 22-kHz USV calls during pre-test and test in the open field under IC optogenetic manipulation.**

**Supplemental Figure S4. Histological representation of IC transfected neurons expressing the fluorescent marker after Arch AAVs injection and optical fiber placement.**
